# *Bmp4* Is Essential for the Formation of the Vestibular Apparatus that Detects Angular Head Movements

**DOI:** 10.1371/journal.pgen.1000050

**Published:** 2008-04-11

**Authors:** Weise Chang, Zhengshi Lin, Holger Kulessa, Jean Hebert, Brigid L. M. Hogan, Doris K. Wu

**Affiliations:** 1 National Institute on Deafness and Other Communication Disorders, NIH, Rockville, Maryland, United States of America; 2 Department of Neuroscience, Albert Einstein College of Medicine, New York, New York, United States of America; 3 Department of Molecular Genetics, Albert Einstein College of Medicine, New York, New York, United States of America; 4 Department of Cell Biology, Duke University Medical Centre, Durham, North Carolina, United States of America; Medical Research Council Human Genetics Unit, United Kingdom

## Abstract

Angular head movements in vertebrates are detected by the three semicircular canals of the inner ear and their associated sensory tissues, the cristae. Bone morphogenetic protein 4 (Bmp4), a member of the Transforming growth factor family (TGF-β), is conservatively expressed in the developing cristae in several species, including zebrafish, frog, chicken, and mouse. Using mouse models in which *Bmp4* is conditionally deleted within the inner ear, as well as chicken models in which Bmp signaling is knocked down specifically in the cristae, we show that *Bmp4* is essential for the formation of all three cristae and their associated canals. Our results indicate that *Bmp4* does not mediate the formation of sensory hair and supporting cells within the cristae by directly regulating genes required for prosensory development in the inner ear such as *Serrate1* (*Jagged1* in mouse), *Fgf10*, and *Sox2*. Instead, Bmp4 most likely mediates crista formation by regulating *Lmo4* and *Msx1* in the sensory region and *Gata3*, *p75Ngfr,* and *Lmo4* in the non-sensory region of the crista, the septum cruciatum. In the canals, *Bmp2* and *Dlx5* are regulated by Bmp4, either directly or indirectly. Mechanisms involved in the formation of sensory organs of the vertebrate inner ear are thought to be analogous to those regulating sensory bristle formation in *Drosophila*. Our results suggest that, in comparison to sensory bristles, crista formation within the inner ear requires an additional step of sensory and non-sensory fate specification.

## Author Summary

Disruption of the sense of balance is highly debilitating, causing vertigo and nausea. Maintenance of proper balance requires sensory inputs from many body parts, including the inner ears and the eyes. Within the inner ear, the vestibular apparatus plays a key role in the sense of balance and is responsible for detecting head orientation and movements. The portion of the vestibular apparatus that detects angular head movements consists of three fluid-filled, semicircular canals oriented at right angles to each other. At one end of each canal is an enlargement that houses the sensory tissue, crista ampullaris, consisting of sensory hair cells and supporting cells. Bone morphogenetic protein 4 (Bmp4), a secreted signaling molecule, is expressed in these sensory regions during development. However, the lack of *Bmp4* in mice affects the formation of not only the sensory regions but also their associated canals. These results demonstrate for the first time that a single gene, *Bmp4*, is required for the formation of the entire sensory apparatus for detecting angular head movements.

## Introduction

**Figure 1:**
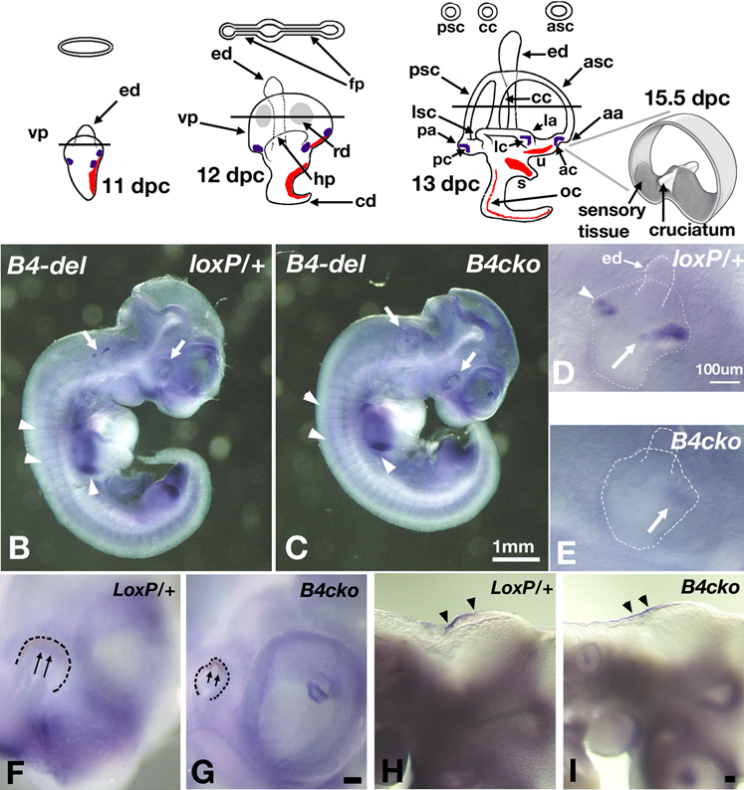
Schematic representations of mouse inner ear development from 11.5 to 13 dpc. (A)Upper panel shows schematic cross-sections through the prospective or definitive anterior and posterior canals at the level of the lines. Blue marks the three *Bmp4*-positive presumptive cristae, while red marks the other three sensory tissues-the maculae utriculi and sacculi, and the organ of Corti. An enlargement of a mature anterior crista at 15.5 dpc or later is shown. (B–I) Inner ear phenotypes of *Bmp4* conditional null embryos. Wholemount in situ hybridization of *Bmp4^loxP/+^* (B,D,F,H) and *Foxg1^cre/+^*; *Bmp4^loxP/Tm1^* (*B4cko*, C,E,G,I) embryos at 10.5 dpc hybridized with *Bmp4* RNA probe specific for exons 3 and 4 (*B4-del*). (B, C) Arrows point to the down-regulation of *Bmp4* expression in the eyes and otocysts of *Foxg1^cre/+^*; *Bmp4^loxP/Tm1^* (C), compared to *Bmp4^loxP/+^* embryos (B). Arrowheads point to unaffected *Bmp4* expression in limb buds and somites. (D) and (E) are higher magnifications of the otocysts shown in (B) and (C), respectively. Arrow and arrowhead in (D) point to *Bmp4* hybridization signals in the anterior streak (encompassing anterior and lateral cristae) and the posterior crista of the otocyst, respectively. An arrow in (E) points to the residual *Bmp4* expression in the anterior streak of *Foxg1^cre/+^*; *Bmp4^loxP/Tm1^* embryos. (F–I) Higher magnifications of *Bmp4* expression domains in the eyes (F, G) and hindbrain (H,I) in *Bmp4^loxP/+^* (F, H) and *Foxg1^cre/+^*; *Bmp4^loxP/Tm1^* (G, I) embryos. Arrows point to the reduction of *Bmp4* expression, and the malformation of the eyes, whereas arrowheads point to the normal *Bmp4* expression in the hindbrain. Scale bar in (C) applies to (B); scale bars in (D), (G) and (I) equal 100μm and apply to (E), (F), and (H), respectively. Abbrevations: aa, anterior ampulla; ac, anterior crista; asc, anterior semicircular canal; cc, common crus; cd, cochlear duct; ed, endolymphatic duct; fp, fusion plate; hp, horizontal canal pouch; la, lateral ampulla; lc, lateral crista; lsc, lateral semicircular canal; oc, organ of Corti; pa, posterior ampulla; pc, posterior crista; psc, posterior semicircular canal; rd, resorption domain; s, saccule; u, utricle; vp, vertical canal pouch.

The ability to detect angular head movements in vertebrates lies within the vestibular apparatus of the inner ear [1]–[3]. This portion of the apparatus consists of three fluid-filled semicircular canals (anterior, lateral and posterior) that are oriented in nearly orthogonal planes (Figure 1A). Each canal contains an enlarged ampulla that houses the sensory tissue, the crista ampullaris, consisting of sensory hair cells and supporting cells. Within the anterior and posterior cristae of many species such as birds and mice, there is a non-sensory structure, the septum cruciatum, which divides the sensory region into two equal halves [4],[5]. This septum cruciatum is not present in the lateral crista. Other vestibular sensory organs that are common among all vertebrates are the maculae of the utricle and saccule, which detect head position and linear acceleration. In fishes, the macula of the saccule is used for hearing as well [6].

All the sensory patches within the vertebrate inner ear including the presumptive cristae are thought to arise from a common prosensory (neural/sensory competent) region at the otic placode and otocyst stages (Figure 1A, red and blue; [7],[8]). This prosensory domain also gives rise to the neurons that innervate various sensory patches of the inner ear. The three semicircular canals are non-sensory structures derived from two epithelial outpouches of the developing otocyst. The vertical outpouch gives rise to the anterior and posterior canals that are joined by the common crus, whereas the horizontal outpouch gives rise to the lateral canal. In the mouse, the morphogenesis of this apparatus starts around 10.5 days post coitum (dpc) and is completed by 13 dpc [9]. In chicken, it starts at embryonic day 3.5 (E3.5) and is completed by E7 [10].

Multiple factors are thought to regulate the formation of the vestibular apparatus [11]–[15]. For example, Wnt signaling from the dorsal hindbrain is required for the normal patterning of the vestibular structures [12]. Within the inner ear, members of two homeobox containing gene families, *Dlx* and *Hmx*, have also been implicated [13],[15]. The deletion of one or more members of these gene families results in the lack of canal and crista formation. Notably, the lack of *Wnt*, *Dlx*, or *Hmx* gene functions all result in an early disorganization or absence of *Bmp4* expression within the presumptive cristae [12],[13],[15],[16].

The expression of *Bmp4* in the presumptive cristae is conserved among several vertebrate species including the zebrafish, frog, chicken, and mouse (Figure 1D; [9], [17]–[19]). Studies in the chicken have shown that the formation of the semicircular canals and cristae is blocked by exogenous Noggin, a Bmp antagonist [20],[21]. However, specific roles for *Bmp4* in inner ear development cannot be extrapolated unambiguously from these results because other *Bmp* genes are also expressed in the developing inner ear, including *Bmp2* and *Bmp7* [22]. In the mouse, the role of *Bmp4* in inner ear development cannot be directly demonstrated either, since *Bmp4* null mutant embryos die before significant vestibular development [23]. More recent in vitro experiments of gain- and loss-of Bmp functions in chicken embryos also yielded conflicting results regarding the role of Bmp4 in hair cell formation [24],[25]. To overcome these problems, we have exploited the cre/lox approach to generate mice with an inner ear specific deletion of *Bmp4*. Furthermore, we address the molecular mechanisms by which Bmp4 mediates its effects on crista formation by over-expressing *Smad6* or *Noggin* in the developing anterior crista to knock down Bmp functions. The combined results from these two species demonstrate that *Bmp4* in the presumptive cristae is required for the formation of the three cristae and their semicircular canals.

## Materials and Methods

### Mouse Strains

The *Bmp4^loxP^* allele was generated by first constructing a targeting vector in which *loxP* sites were inserted in introns 2 and 4 of the *Bmp4* locus, so that cre recombination excises the entire *Bmp4* coding sequence (Figure S1). *Bmp4^Tm1/+^* and *Bmp4^loxP/loxP^* mice were maintained on a Black Swiss background and *Foxg1^cre/+^* mice were maintained on a Swiss Webster background. *Foxg1^cre/+^*; *Bmp4^loxP/Tm1^* embryos were generated by crossing male *Foxg1^cre/+^*; *Bmp4^Tm1/+^* mice with female *Bmp4^loxP/loxP^* mice. For reasons that are unknown, very few *Foxg1^cre/+^*; *Bmp4^loxP/Tm1^* mice were recovered at birth (Table S1). Therefore, all analyses in this study were conducted by 13.5 dpc, an age when the gross patterning of the canals and ampullae is complete. *TgPax2cre*; *Bmp4^loxP/Tm1^* embryos were generated by breeding *TgPax2cre*; *Bmp4^Tm1/+^* with *Bmp4^loxP/loxP^* mice. The generation of *TgPax2cre,* a transgenic mouse strain expressing *cre* under an inner ear specific enhancer of *Pax2*, will be described elsewhere (Douglas Epstein, U. of Pennsylvania). All animal procedures were approved and conducted according to the NIH Animal Use and Care Committee guidelines.

### Chicken Embryos and Procedures

Chicken embryos were staged according to Hamburger and Hamilton [26]. Chicken *Noggin* cDNA [27] was subcloned into pIRES2-EGFP expression vector, in which *Noggin* is driven by the immediate early Cytomegalovirus promoter (Clontech). Chicken *Smad6* cDNA in the pCab-IRES-GFP vector [28] was subcloned into pMES-IRES-GFP expression vector, in which *Smad6* is driven under the chicken β-actin promoter and the immediate early enhancer of Cytomegalovirus [29]. *pSmad6*, *pNoggin* and their respective control vectors at a concentration of 4 to 6 mg/ml were injected into the lumen of chicken otocysts at E3.5. Plasmids were electroporated into the anterior region of the otocyst using a positive and negative electrode flanking the anterior and posterior poles of the otocyst, respectively. Two 50 milli-second pulses at 10 volts were applied using a CUY21 electroporator.

### In Situ Hybridization and Immunostaining

Paint-fill analyses and in situ hybridizations were performed as described [9]. Chicken and mouse RNA probes were prepared as previously described [9], [19], [30]–[32].

Anti-hair cell specific antigen (HCA) antibodies (gift of Guy Richardson) were used at 1:5000 dilution, and staining was performed as previously described [33]. Specimens for antibody staining were fixed overnight at 4°C with 4% paraformaldehyde, except specimens for Msx1/2 and Gata3 staining were fixed for 30 minutes at room temperature. The following antibody dilutions were used: mouse anti-neurofilament (DSHB, 3A2) 1∶2000; mouse anti-Msx1/2 (DSHB, 4G1), 1∶50; rabbit anti-Phospho-Smad1 (gift of Peter ten Dijke), 1∶2000; mouse anti-Sox2 (Chemicon, AB5603), 1∶2000; mouse anti-Gata3 (Santa Cruz, HG3-31), 1∶50; and Goat anti-GFP antibody (GeneTexa, GTX26662), 1∶200. For secondary antibody labeling, species-specific antibodies conjugated with Alexa Fluor 488, 564, or 633 were used at 1∶500 dilution. Incubations for primary and secondary antibodies were carried out at 4°C overnight and at room temperature for 1 hr, respectively. Total number of double-labeled cells for each specimen was scored using a confocal microscope. Since the total number of cells counted per specimen was different, weighted average percentages (wap) were calculated for each treatment to adjust for the variability of sampling size among specimens (http://mathforum.org/library/drmath/view/57605.html). A total of 70 to 190 cells were counted per treatment.

## Results

### Inner Ear Phenotypes of *Bmp4* Conditional Knockout Embryos

To generate conditional *Bmp4* null embryos, we used three different mouse lines. The first, *Foxg1^cre/+^*, was made by inserting *cre* into the endogenous *Foxg1* gene which is expressed in tissues such as the embryonic otocysts, eyes, and foregut [34]. The tissue specific recombination activity of this *cre* allele has been demonstrated by crossing *Foxg1^cre/+^* mice with the *Rosa26R* reporter line [34]. *Bmp4^Tm1^* is a null allele of *Bmp4* [23], whereas the *Bmp4^loxP^* conditional allele was generated as described (Figure S1). *Foxg1^cre/+^*; *Bmp4^loxP/Tm1^* embryos were obtained at the expected frequency from crossing *Foxg1^cre/+^*; *Bmp4^Tm1/+^* mice with *Bmp4^loxP/loxP^* mice. Based on morphologies, they can be grouped into three classes: (1) embryos that are severely delayed in development, (2) embryos with eye malformations that are either normal or slightly smaller in their body size, and (3) embryos that are morphologically indistinguishable from *Bmp4^loxP/+^* littermates (Table S1). Only the latter two classes were included in subsequent studies.

We evaluated the tissue specificity of *Bmp4* deletion in the *Foxg1^cre/+^*; *Bmp4^loxP/Tm1^* embryos between 9.5 to 10.5 dpc (n = 21) using an RNA probe (*B4-del*) generated against exons 3 and 4 of *Bmp4*. Half of the embryos analyzed at 9.5 dpc displayed abnormal *Bmp4* expression patterns (n = 4/8). By 10.5 dpc, a higher percentage of *Foxg1^cre/+^*; *Bmp4^loxP/Tm1^* embryos show no or reduced *Bmp4* expression in tissues such as the eyes and otocysts where *Foxg1* is normally transcribed (Figure 1B–1G, arrows; n = 11/13). Significantly, expression patterns are normal in tissues where *Foxg1* is not expressed such as the roof of the hindbrain, somites and limb buds (Figure 1B,1C,1H, and 1I, arrowheads). Some of the eleven embryos that display tissue-specific reduction in *Bmp4* expression and eye malformations were also slightly smaller in body size (n = 3).

In a normal otocyst, *Bmp4* is transcribed in an anterior streak of tissue and a posterior focus (Figure 1D; [9]). The anterior streak encompasses the presumptive anterior and lateral cristae (Figure 1D, arrow) and later splits to form two separate entities [9], whereas the posterior focus demarcates the location of the posterior crista (Figure 1D, arrowhead). Among the 11 affected *Foxg1^cre/+^*; *Bmp4^loxP/Tm1^* 10.5 dpc embryos, *Bmp4* transcripts are absent from the posterior region of the otocyst and are either absent or reduced in the anterior (Figure 1E, arrow). Similar results were obtained from affected *Foxg1^cre/+^*; *Bmp4^loxP/Tm1^* specimens at 11.5 dpc (see below). By this age, *Bmp4* is also expressed in the non-sensory region of the growing cochlear duct [9]. Despite the seemingly ubiquitous cre activity in the otocysts of the cre reporter mice [34], *Bmp4* expression in the cochlear duct appears normal in all of the *Foxg1^cre/+^*; *Bmp4^loxP/Tm1^* specimens examined (data not shown).

### Paint-Filled Analyses of *Bmp4* Conditional Knockout Inner Ears

**Figure 2:**
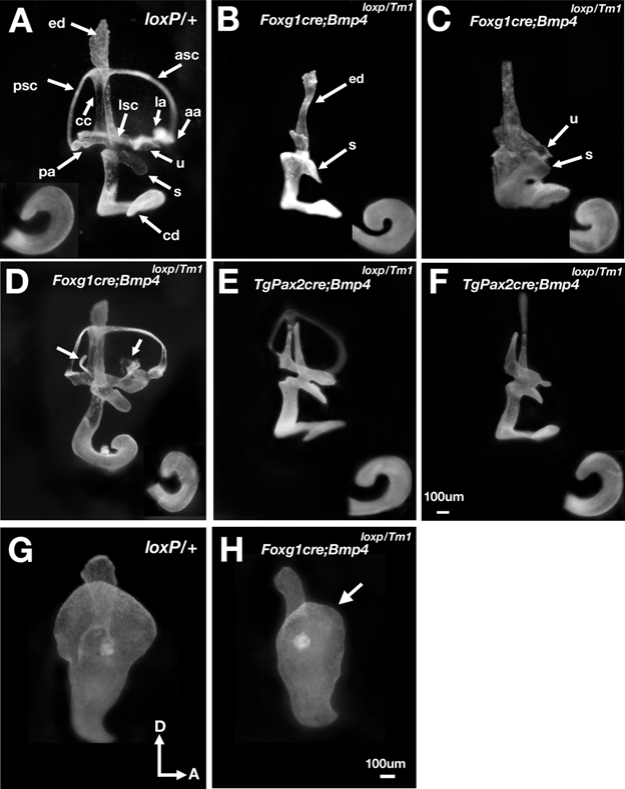
Inner ear analyses of *Bmp4* conditional null embryos. Paint-filled inner ears of control *Bmp4^loxP/+^* (A,G), *Foxg1^cre/+^*; *Bmp4^loxP/Tm1^* (B–D,H) and *TgPax2cre*; *Bmp4^loxP/Tm1^* (E,F) embryos at 11.5 (G,H) and 13.5 dpc (A–F). Inserts in (A)–(F) are ventral views of the cochlear duct. The most malformed inner ears of *Foxg1^cre/+^*; *Bmp4^loxP/Tm1^* embryos are shown in (B) and (C), compared to controls (A). In (D), the inner ear is normal except for truncation of the lateral canal (arrows). A mildly (E) and more severely (F) affected inner ear of *TgPax2cre*; *Bmp4^loxP/Tm1^* embryos. Inner ears of *Bmp4^loxP/+^* (G) and (H) *Foxg1^cre/+^*; *Bmp4^loxP/Tm1^* embryos at 11.5 dpc. Arrow in (H) points to the smaller canal pouch in *Foxg1^cre/+^*; *Bmp4^loxP/Tm1^* embryos. Orientations in (G) apply to all panels. Scale bars in (F) and (H) apply to (A–E) and (G), respectively.

The gross anatomy of the *Foxg1^cre/+^*; *Bmp4^loxP/Tm1^* inner ears at 13.5 dpc was examined by paint filling the membranous labyrinth. Consistent with the variable *Bmp4* expression patterns, the paint-filled *Foxg1^cre/+^*; *Bmp4^loxP/Tm1^* specimens also show a range of inner ear phenotypes (Figure 2A–2D). In the most severe cases, there is no discernible ampulla or semicircular canal, and the utricle and saccule are malformed. Only an intact endolymphatic duct is evident in the dorsal region of the inner ear (Figure 2B and 2C; n = 8/14). The remaining specimens are either indistinguishable from *Bmp4^loxP/+^* (n = 3/14) embryos, or display only a lateral canal truncation (Figure 2D; n = 3/14). A percentage of the *Bmp4^loxP/Tm1^* also display similar defects in the lateral canal (n = 5/10). Therefore, this milder phenotype observed in *Foxg1^cre/+^*; *Bmp4^loxP/Tm1^* embryos is probably due to insufficiency of *Bmp4* caused by the presence of both of the *Tm1* and the un-recombined floxed *Bmp4* allele rather than an incomplete penetrance of the cre activity. Cochlear ducts of *Foxg1^cre/+^*; *Bmp4^loxP/Tm1^* embryos show some variability in length (Figure 2B–2D). We attributed this variability to a slight difference in staging or global growth defects of the ear.

We also conditionally deleted *Bmp4* in the inner ear using a transgenic mouse strain, *TgPax2cre*. The inner ear phenotypes obtained using this *cre* strain are also variable. Ten out of 15 *TgPax2cre*; *Bmp4^loxP/Tm1^* specimens have inner ear defects. Those with a milder phenotype show defects in the three ampullae and canals, in addition to lateral canal truncation (Figure 2E; n = 5). The more severe phenotypes include utricle and saccule malformations (Figure 2F; n = 5). Similar to the *Foxg1^cre/+^*; *Bmp4^loxP/Tm1^* inner ears, the cochlear duct is relatively normal, consistent with the presence of *Bmp4* expression in this region (data not shown). Taken together, inner ear-specific deletion of *Bmp4* using two independent *cre* lines indicates that *Bmp4* is required for the formation of the three cristae and semicircular canals, and possibly the utricle and saccule.

Some of the *Bmp4^Tm1/loxP^* embryos generated by breeding *TgPax2cre*; *Bmp4^+/Tm1^* with *Bmp4^loxP/loxP^* mice also display lateral canal truncation as well (n = 4/7), suggesting that a combination of *Tm1* and *loxP* alleles can generate hypomorphs depending on the genetic background. Notably, our *Bmp4^Tm1/+^* mice in Black Swiss background do not circle but a small percentage of *Bmp4* heterozygous mice in C57BL/6 background do [35].

### Gene Expression Analyses of *Foxg1^cre/+^; Bmp4^loxP/Tm1^* Embryos

**Figure 3:**
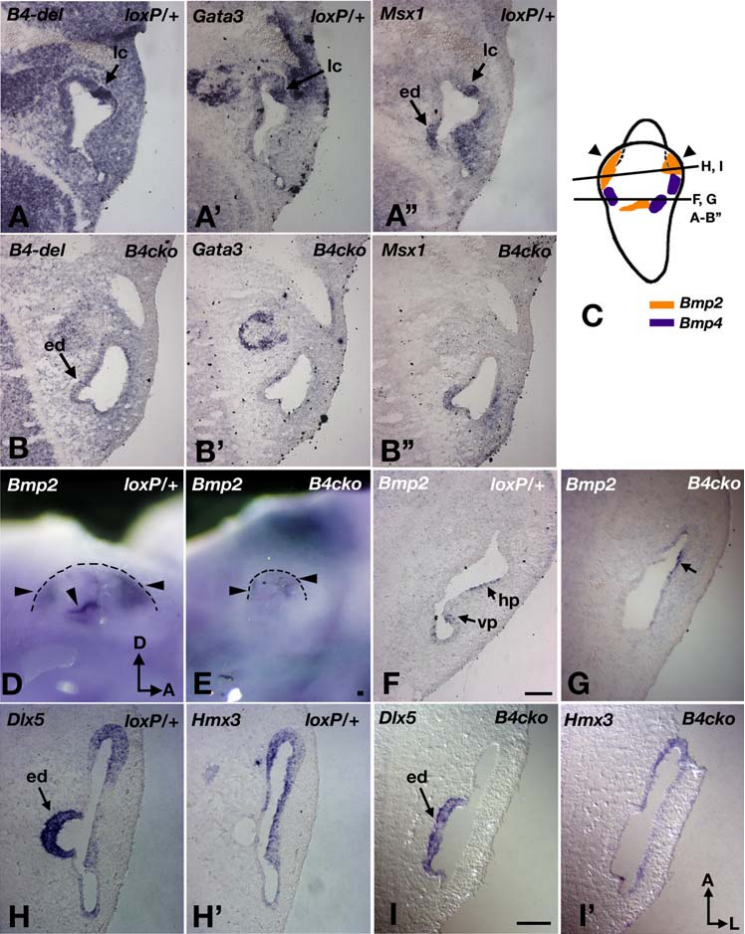
Gene expression analyses of *Foxg1^cre/+^*; *Bmp4^loxP/Tm^* inner ears at 11.5 dpc. (A–A') Adjacent sections of a *Bmp4^loxP/+^* control showing the *Bmp4*-positive lateral crista region (A, lc), which is also positive for *Gata3* (A') and *Msx1* (A”). (B,B',B”) Adjacent sections of a *Foxg1^cre/+^*; *Bmp4^loxP/Tm1^* (*B4cko*) embryo showing the lack of crista-associated expression of *Bmp4* (B), *Gata3* (B') and *Msx1* (B”). (C) A schematic diagram showing the expression domains of *Bmp2* and *Bmp4* in the canal pouch at 11.5 dpc, and the approximate level of section for each panel. (D–G) Wholemount (D,E) and section (F,G) in situ hybridization showing the reduction of *Bmp2* expression in the canal pouch (outlined in D, E) of *Foxg1^cre/+^*; *Bmp4^loxP/Tm1^* (E,G), compared to *Bmp4^loxP/+^* (D,F) inner ears. (F) *Bmp2* expression is associated with the prospective posterior and lateral canals (vp and hp) in *Bmp4^loxP/+^* embryos but only in the anterior region of the canal pouch in *Foxg1^cre/+^*; *Bmp4^loxP/Tm1^* embryos (G, arrow) where residual *Bmp4* expression is sometimes present. (H–I') *Dlx5* (H, I) and *Hmx3* (H',I') expression domains in the canal pouch of *Bmp4^loxP/+^* (H,H') and *Foxg1^cre/+^*; *Bmp4^loxP/Tm1^* (I,I') embryos. The endolymphatic duct (ed) is *Dlx5*-positive (H,I) and *Hmx3*-negative (H',I'). Canal pouches of *Foxg1^cre/+^*; *Bmp4^loxP/Tm1^* inner ears are *Dlx5* negative (I) and *Hmx3* positive (I'). Orientations: A, anterior; L, lateral. Orientations in (I') apply to all panels except (D) and (E). Scale bars = 100 μm. Scale bar in (F) applies to (A–B) and (G); scale bars in (E) and (I) apply to (D) and (H–I'), respectively.

Analysis of paint-filled ears of younger embryos indicates that the vestibular defects in *Foxg1^cre/+^*; *Bmp4^loxP/Tm1^* are already apparent at 11.5 dpc (Figure 2G and 2H). To better understand the underlying molecular mechanisms of the phenotypes, we first investigated the expression patterns of a number of genes associated with the prospective cristae such as *Fgf10*, *Gata3*, *Jag1*, *Lmo4*, *Msx1*, and *Sox2,* in *Foxg1^cre/+^*; *Bmp4^loxP/Tm1^* embryos. At 11.5 dpc, the expression domains of these genes normally overlap with that of *Bmp4* in the presumptive cristae (Figure 3A; data not shown). Conditional mutant ears that have smaller canal pouches compared to those of *Bmp4^loxP/+^* embryos are usually devoid of *Bmp4* expression and concomitant loss of other early crista-associated markers as well (Figure 3B–B”, n = 28/42 ears). Conversely, in all conditional mutants with residual *Bmp4* expression in the anterior region (n = 13/42), other crista markers are also present.

Lack of *Bmp4* expression in the inner ear also resulted in the absence of semicircular canal formation. The overall size of the vertical canal pouch is usually smaller than normal, particularly in the posterior region (Figure 2G and 2H). This finding is in agreement with the observation that *Bmp4* expression is lost most consistently in the posterior crista of *Foxg1^cre/+^*; *Bmp4^loxP/Tm1^* ears. *Bmp2* has been implicated in canal formation in the chicken inner ear [36], and its expression pattern in the canal pouches of mice is similar to that of chickens (Figure 3D and 3F). At 11.5 dpc, *Bmp2* expression in *Foxg1^cre/+^*; *Bmp4^loxP/Tm1^* inner ears is often reduced and sometimes absent in the canal pouches (Figure 3E; arrowheads, n = 8; Figure 3G; n = 4/6, missing posterior signal).

Other genes such as *Dlx5*, *Hmx2*, and *Hmx3* have also been implicated in canal development. *Dlx5* and *Hmx* are expressed in the otic placode and later in the entire canal pouch (Figure 3H–H'; [37]–[40]). In *Foxg1^cre/+^*; *Bmp4^loxP/Tm1^* embryos with affected inner ears, *Dlx5* expression is down-regulated in the canal pouch, but the expression of *Hmx3* is unaltered at least up to 11.5 dpc (Figure 3I–I'; n = 4). In contrast, *Dlx5* expression in the endolymphatic duct is normal (Figure 3I). These results suggest that *Bmp4* is required for the maintenance of *Dlx5* expression only in the canal pouch, and that the regulation of *Hmx3* in the canal pouch is independent of *Bmp4* and *Dlx5*.

### Gene Expression Patterns in the Differentiating Chicken Crista

**Figure 4:**
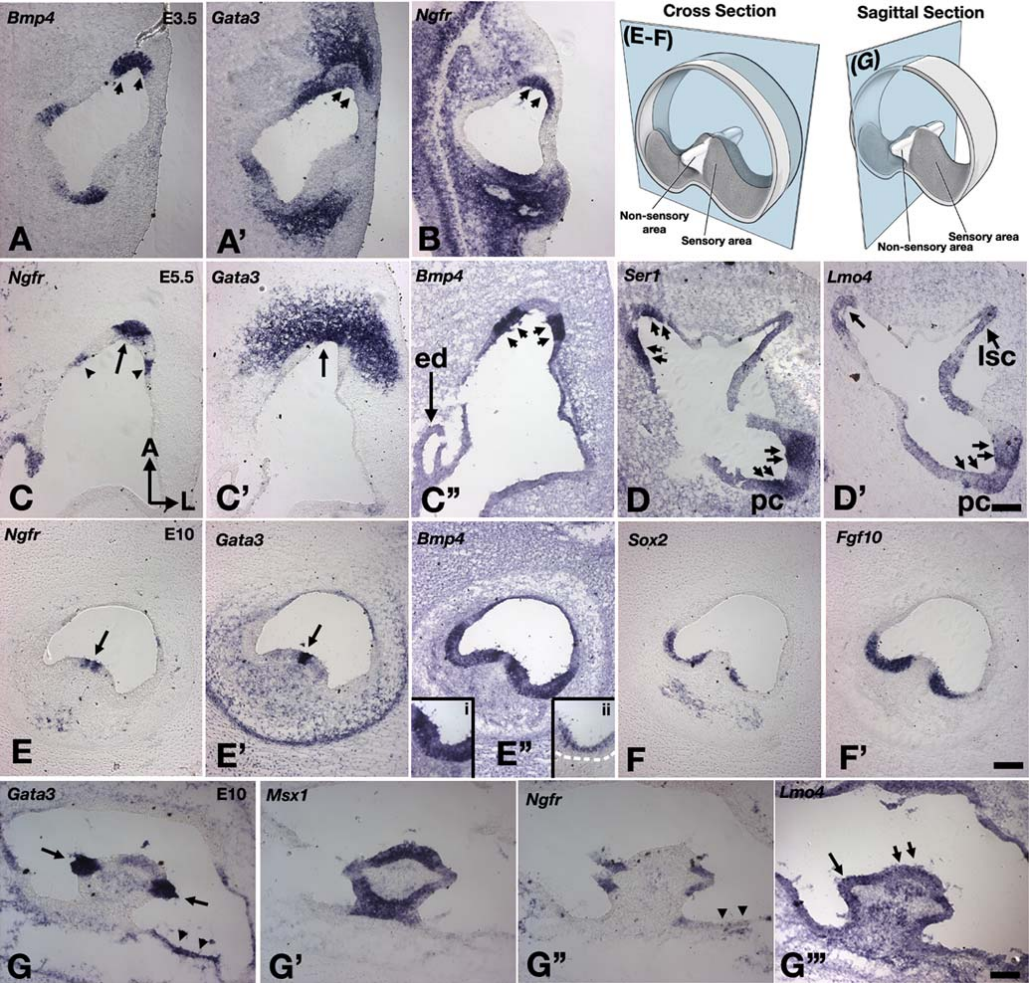
Expression patterns of crista-associated genes during differentiation. Sections of developing chicken cristae at E3.5 (A,B), E5.5 (C,D) and E10 (E–G). (A, A') Adjacent sections showing the co-expression of *Bmp4* (A) with *Gata3* (A') and *p75Ngfr* (B) in the anterior crista region at E3.5. (C–C”) Adjacent sections showing expression patterns of *p75Ngfr* (C) and *Gata3* (C') largely non-overlapping with the two *Bmp4-*positive regions (C”). *Gata3* is also expressed in the mesenchymal region surrounding the crista. (D) *Ser1* expression pattern in the developing crista is similar to that of *Bmp4* (C”), in two separate domains (double arrows), whereas (D') *Lmo4* is expressed in the *Ser1*-positive regions (pc, double arrows) as well as in the area between the two *Ser1*-positive regions (arrow). (E–F) Cross- and (G) sagittal-sections of the developing crista at E10. (E”) *Bmp4*, (F) *Sox2*, (F') *Fgf10*, and (G') *Msx1* are expressed in the sensory region of the developing crista, whereas (E) *p75Ngfr* and (E') *Gata3* are expressed in the non-sensory, cruciatum region in the center of the crista. Inserts (i) and (ii) in (E”) are higher magnifications of a sensory region in (E”) showing the *Bmp4* expression domain spanning the entire epithelium and the *Bdnf* domain only located apically in the sensory hair cells, respectively. The dotted line in insert (ii) marks the base of the sensory epithelium. The expression domain of *p75Ngfr* appears to surround the *Gata3*-positive region in the cruciatum (E, E'; G, G”). Moreover, both *Gata3* and *p75Ngfr* are expressed in the transitional zone of the developing crista (G, G”; arrowheads). The *p75Ngfr* expression in the transitional zone is already apparent at E5.5 (Figure 4C, arrowheads). (G”’) *Lmo4* is expressed in both the sensory (small arrows) and cruciatum (arrow) region of the developing crista. Orientations in (C) apply to (A–D'). (E–F') and (G'–G”’) are cross- and sagittal-sections of the anterior crista at E10, respectively. Scale bars = 100 μm. Scale bars in (D'), (F') and (G”’) apply to (A–D), (E–F) and (G–G”), respectively.

Our results suggest that absence of *Bmp4* affects the expression patterns of many genes in the presumptive cristae of mice. However, it is not clear whether these changes are direct or indirect due to the loss of sensory tissues in the conditional mutants. To address this question, we analyzed the short-term effects of down-regulating Bmp signaling on seven known crista-associated genes-*Fgf10*, *Gata3, Lmo4, Msx1, p75Ngfr, Ser1*, and *Sox2*-in chicken inner ears. First, we examined in more detail the expression profiles of these genes during normal crista development (Figure 4). In a mature anterior or posterior crista, the sensory patch is saddle-shaped, consisting of sensory hair cells and supporting cells. A non-sensory region, the septum cruciatum, is located in the middle of the saddle (Figure 4, schematic diagrams). Initially, the expression pattern of each of the seven investigated genes largely overlaps with the expression domain of *Bmp4* in the presumptive anterior or posterior crista (Figure 4A, A', B; [30]; [19]; data not shown). After E3.5, the expression patterns of these genes start to segregate (Figure 4C–C”, D–D'). At E5.5, genes such as *Bmp4*, *Sox2*, *Ser1*, and *Fgf10* are expressed in two separate domains associated with the sensory patches (Figure 4C”, D, double arrows; data not shown). In between the two sensory patches is the *p75Ngfr-* and *Gata3*-positive region that eventually develops into the septum cruciatum (Figure 4C–C”, arrow). In contrast, *Lmo4* is expressed in both sensory (Figure 4D', double arrows in pc) and non-sensory regions (Figure 4D', arrow) of the presumptive crista, and this expression pattern is maintained at E10 (Figure 4G”’). By E10, *Bmp4*, *Fgf10, Msx1, Sox2*, and *Ser1* are associated with supporting cells of the sensory region (Figure 4E”, F, F', G'). The expression patterns of these genes are qualitatively different from that of *Bdnf*, which is associated with sensory hair cells (Figure 4E”, insert ii). *Gata3* and *p75Ngfr* expression domains remain outside of the sensory tissue proper, in the septum cruciatum (Figure 4E–E', G, G”, arrows; [32]) as well as in the transitional zone beyond the crista (Figure 4G, G”, arrowheads).

In summary, our analyses indicated that while most of the crista-associated genes initially overlap in their expression domains within the presumptive anterior and posterior cristae, their expression patterns segregate into either sensory and/or non-sensory regions of the crista as development proceeds.

### Genes Affected by Down-Regulation of Bmp Signal Transduction in the Crista

**Figure 5:**
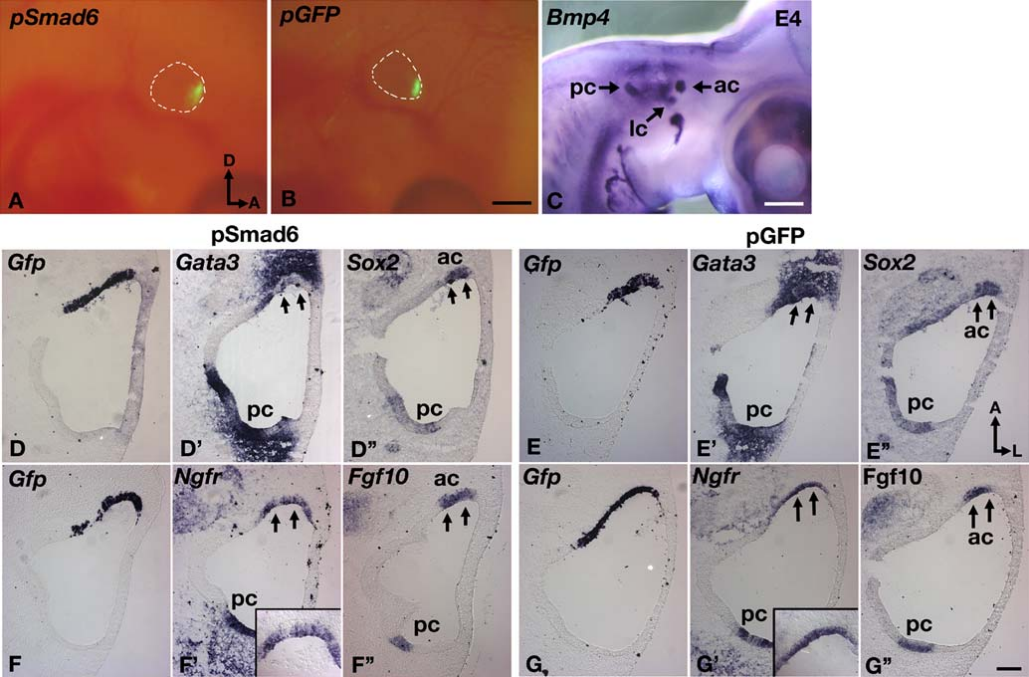
Ectopic expession of *Smad6* down-regulates crista-associated genes in chicken inner ears. (A–C) Whole mount embryos at E4 showing GFP expression in the targeted anterior crista region 14 hrs after electroporation with *pSmad6* (A) or *pGfp* (B) plasmids. (C) *Bmp4* expression in the cristae. (D–G”) Sections of inner ears electroporated with *pSmad6* (D,F) or *pGfp* (E,G) plasmids at E3.5 and harvested 14 hrs after electroporation. (D–D”) Adjacent sections probed for *Gfp* (D), *Gata3* (D') and *Sox2* (D”) transcripts. Within the electroporated anterior crista region (D), *Gata3* expression is down-regulated (D', arrows), whereas *Sox2* expression is unaffected (D”). (F–F”) Down-regulation of *p75Ngfr* (F') in the electroporated region (F), but expression of *Fgf10* is not affected (F”, arrows). None of these gene expression patterns are affected in controls electroporated with *pGfp* (E, G). Inserts in (F') and (G') are higher magnifications of the anterior crista. Scale bars in (B) and (C) equal 1 mm and scale bar in (B) applies to (A). Scale bar in (G”) equals 100 μm and applies to (D–G').

Next, we investigated whether the expression of each of these crista-associated genes is affected by down-regulation of Bmp signaling. Vectors (*pSmad6* and *pNoggin*) encoding *Smad6* or *Noggin* translationally coupled to GFP were electroporated into the developing anterior crista region in ovo at E3.5, a time when these crista-associated genes are co-expressed in the presumptive crista. Smad6 is an intracellular inhibitor that competes with Smad4 for binding to phosphorylated Smad1/Smad5/Smad8 proteins, thus preventing their subsequent translocation to the nucleus and activation of Bmp target genes [41]. Ectopic *Smad6* expression has been used successfully to address the roles of Bmps in neural induction and placode formation [28],[42]. Figure 5A and B illustrate Gfp signals in the *Bmp4*-positive anterior crista region (Figure 5C) within 14 hrs after electroporation with *pSmad6* and *pGfp*, respectively.

**Figure 6:**
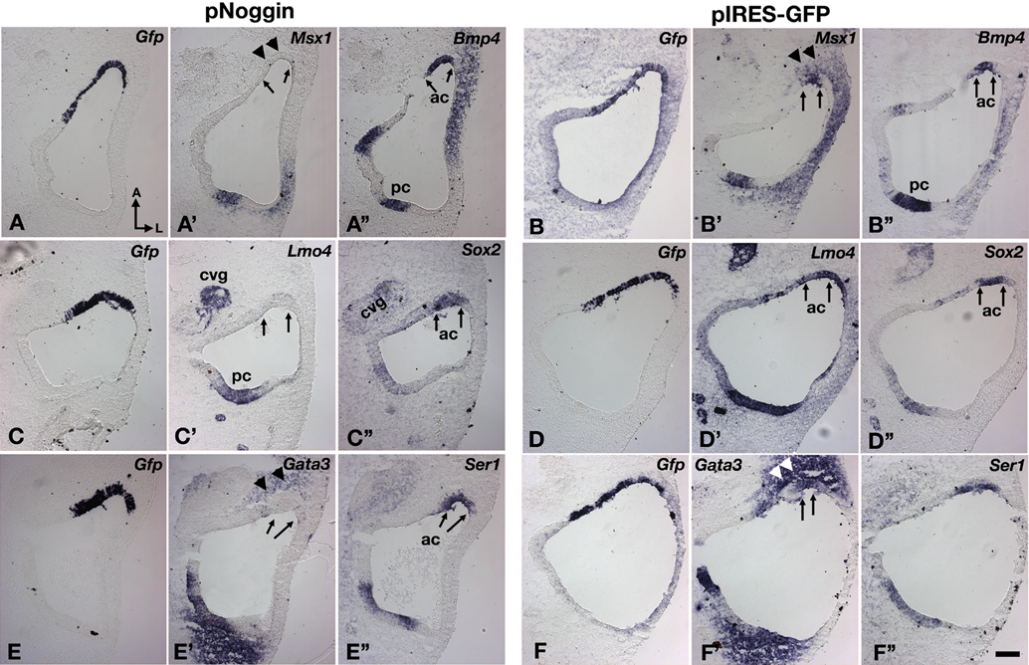
Ectopic expression of *Noggin* down-regulates crista-associated genes in chicken inner ears. Inner ears were electroporated with *pNoggin* (A,C,E) and p*IRES*-*Gfp* (B,D,F) at E3.5 and harvested 14 hrs later. (A–A”) Adjacent sections probed for *Gfp* (A), *Msx1* (A'), and *Bmp4* (A”) transcripts. *Msx1* (A') expression is abolished in the electoporated (A), *Bmp4*-positive anterior crista region (A”, ac), whereas *Bmp4* expression is not affected (A”). *Msx1* expression is reduced in the mesenchymal region (A', arrowheads). (C–C”) Adjacent sections showing the absence of *Lmo4* (C') in the electroporated (C), *Sox2*-positive anterior crista region (C”, arrows). (E–E”) Adjacent sections probed for *Gfp* (E), *Gata3* (E'), and *Ser1* (E”) transcripts. (E') *Gata3* expression is down-regulated in the anterior crista (arrows) as well as the surrounding mesenchyme (arrowheads), but *Ser1* expression is not changed (E”). (B,D,F) None of these gene expression patterns are affected in specimens electroporated with the *pIRES-Gfp*. Abbreviations: cvg, cochleovestibular ganglion. Scale bar in (F”) equals 100 μm and applies to all panels.

Electroporation of *pSmad6* results in the down-regulation of genes that are eventually associated with the non-sensory, septum cruciatum, such as *Gata3* (Figure 5D'; n = 10/10) and *p75Ngfr* (Figure 5F'; n = 10/13), whereas expression levels of genes associated with the sensory regions such as *Sox2* (n = 0/6), *Fgf10* (n = 0/6), *Lmo4* (n = 0/9) and *Ser1* (n = 0/5) are not affected (Figure 5D”,F”, and data not shown). Electroporation of a control vector expressing *Gfp* alone does not result in gene expression changes in most cases (Figure 5E–E”,G–G”; n = 42/44). Down-regulation of *Msx1* in response to *pSmad6* is variable (n = 7/14; data not shown), but quite consistently seen in response to *pNoggin* (Figure 6A'; n = 6/6). The expression of *Lmo4,* which is associated with both sensory and non-sensory regions, is down-regulated by *pNoggin* (Figure 6C'; n = 11/11), but this is not observed with *pSmad6* (n = 9; data not shown). Since Noggin is a secreted molecule, down-regulation of *Gata3* and *Msx1* in the mesenchyme near the site of electroporation is also observed (Figure 6A',B',E',F', double arrowheads; n = 6/6). Genes that are not down-regulated by *pSmad6,* such as *Bmp4, Fgf10*, *Ser1* and *Sox2,* remain unaffected by *pNoggin* treatments (Figure 6A”,C”,E”; data not shown). Electroporation of a control vector, *pIRES-Gfp*, usually causes no change in these gene expression patterns (Figure 6B, 6D, 6F; n = 26/28).

### Blocking Bmp Signaling Down-Regulates Msx and Gata3 Immunoreactivities in the Crista

**Figure 7:**
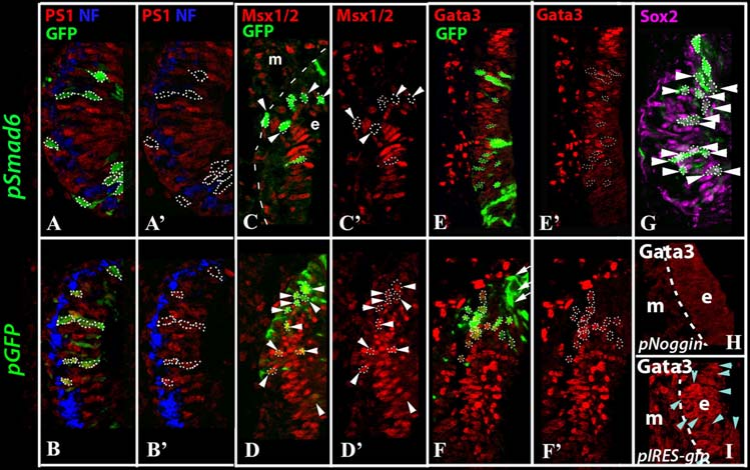
Down-regulation of phosphosmad1, Msx1 and Gata3 immunoreactivities following *pSmad6* and *pNoggin* electroporations. Sections of inner ears electroporated with *pSmad6* (A,C,E,G), *pGfp* (B, D, F), *pNoggin* (H), and *pIRES-Gfp* (I) at E3.5 and harvested 14 hrs later. (A, A') A section stained with anti-phosphosmad1 (PS1, red), anti-GFP (green), and anti-neurofilament (blue) antibodies. Cells electroporated with *pSmad6* (outlined) are GFP-positive (A) and PS1-negative (A'). The neurofilament staining identifies the presumptive crista region. (B, B') A section from an inner ear electroporated with *pGfp* showing the GFP-positive cells (B) are also positive for PS1 staining (B'). (C-D) *pSmad6*-positive cells (C, arrowheads) are negative for anti-Msx staining, whereas GFP-control cells (D, arrowheads) are positive for Msx immunoreactivity (D'). (E–F') *pSmad6*-positive cells in (E) are negative for Gata3 (E'), but GFP-control cells (F) are Gata3 positive (F'). Arrows in (F) point to GFP-positive cells outside of the crista region. (G) *pSmad6*-positive cells are positive for Sox2 staining (arrowheads). (H, I) A *pNoggin*-treated section (H) showing the absence of Gata3 staining in both the epithelium (e) and mesenchyme (m), whereas Gata3 staining is normal in *pIRES-Gfp* treated specimen (I). Blue arrowheads point to cells that are GFP positive (data not shown).

The changes in gene expression were verified at the protein level by double staining the electroporated cells for GFP and translated gene products. The levels of phosphosmad1 were used to evaluate the effects of Smad6 inhibition on Bmp signal transduction. Cells electroporated with *pSmad6* show a down-regulation of phosphosmad1 staining (Figure 7A,A'; wap = 98%, n = 7; see Materials and Methods), whereas cells electroporated with *pGfp* do not (Figure 7B; wap = 23%, n = 7). Moreover, *pSmad6*-electroporated cells also show a down-regulation of Msx (Figure 7C,C'; wap = 94%, n = 4) and Gata3 immunoreactivities (Figure 7E, E'; wap = 87%, n = 8), whereas Sox2 levels are barely affected (Figure 7G; wap = 2.2%, n = 8). Down-regulation of Msx (Figure 7D,D'; wap = 8.6%, n = 5), Gata3 (Figure 7F,F'; wap = 0%, n = 5) and Sox2 (data not shown, wap = 11%, n = 4) immunoreactivities are minimal in cells electroporated with *pGfp*. Similar results are observed with *pNoggin*, except down-regulation of Gata3 staining is also observed in the mesenchyme (Figure 7H; n = 6), whereas Gata3 expression is normal in specimens electroporated with the control plasmid, *pIRES-Gfp* (Figure 7I; n = 6). Taken together, our results suggest that the down-regulation of Bmp signal transduction appears to preferentially affect genes associated with non-sensory rather than sensory region of the crista.

### Inner Ear Phenotypes after Down-Regulation of Bmp Signal Transduction

**Figure 8:**
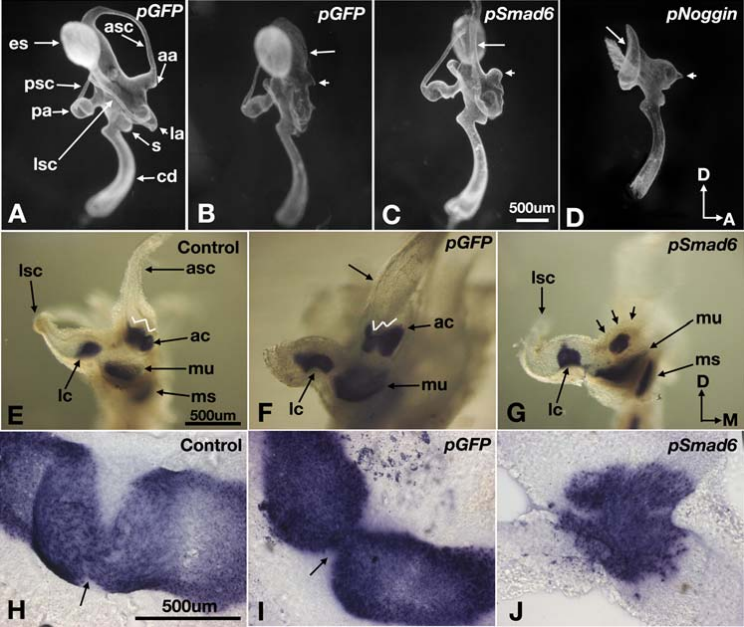
* pSmad6* and *pNoggin*-induced inner ear phenotypes. Paint-filled inner ears at E7 after electroporation with *pGfp* (A, B), *pSmad6* (C), or *pNoggin* (D) at E3.5. Inner ears electroporated with *pGfp* are either normal (A), or show non-resorption of the anterior canal (B, arrow) and absence of a distinct anterior ampulla (B, small arrow). (C) A *pSmad6*-treated inner ear showing a malformed anterior ampulla (small arrow) and absence of the anterior canal. The common crus is wider than normal (arrow). (D) A *pNoggin*-treated inner ear showing the absence of the three ampullae, anterior and lateral canals. The posterior fusion plate is not resorbed (arrow). (E–G) Anti-HCA staining of partially dissected inner ear of controls (E) or inner ears electroporated with *pGfp* (F) or *pSmad6* (G) at E3.5 and harvested at E8.5. (E) Anterior crista shows a typical saddle or W-shaped pattern with anti-HCA staining. (F) *pGfp*-treated inner ear with a canal pouch that is not resorbed (arrow), but the anterior crista appears normal. (G) *pSmad6*-treated inner ear showing a malformed and reduced anterior crista and no anterior canal. Arrows point to the outline of the ampulla. (H, I, J) Flattened anterior cristae from (E, F, G), respectively. Arrows in (H) and (I) point to the location of the septum cruciatum, and punctate staining represents stereocilia bundles on top of the sensory hair cells. Orientations: M, medial. Scale bars in (C), (E), and (H) apply to (A–D), (F–G), and (I–J), respectively.

To investigate whether the knock down of Bmp signal transduction has a long-term effect on crista or canal formation, we harvested some electroporated embryos at E7 and processed them for paint-fill analyses or at E8.5 for sensory hair cell staining using anti-HCA antibody. More than half of the inner ears electroporated with *pGfp* have normal canals (Figure 8A; n = 12/21), and the rest show non-resorption of the anterior canal (Figure 8B,F, arrow; n = 9/21). However, specimens in which the canal pouch fails to resorb, the anterior crista is usually normal showing a saddle-shaped pattern with anti-HCA staining (Figure 8F, I; n = 4/5), similar to controls (Figure 8E, H). Most of the *pSmad6* electroporated specimens either lack the anterior canal or show a canal pouch that is not resorbed (Figure 8C; n = 15/19), and the anterior ampulla is malformed (Figure 8C,G, small arrows; n = 15/19). Within the ampulla, the crista is usually much smaller in size, lacks the cruciatum, and thus lacks the saddle- or W-shaped staining pattern (Figure 8G; n = 10/12). However, some sensory hair cells remain within the malformed cristae based on the punctate staining pattern with anti-HCA antibodies (Figure 8J). These results indicate that down-regulating Bmp signal transduction in the presumptive anterior crista cell-autonomously causes patterning defects in the crista. Inner ears electroporated with *pNoggin* instead of *pSmad6* show a much more severe phenotype involving all three canals and ampullae (Figure 8D; n = 7/8).

The high percentages of specimens with canal defects in the *pGfp* specimens suggest that canal formation is particularly sensitive to electroporation. Furthermore, since the electroporated region often includes some of the canal pouch epithelium, the canal phenotypes observed in *pSmad6* and *pNoggin* specimens could be due to a direct down-regulation of Bmp2 signaling, originating within the canal epithelium [36], rather than down-regulation of Bmp4 signaling generated from the crista.

## Discussion

### Cell Fate Specification in the Crista

**Figure 9:**
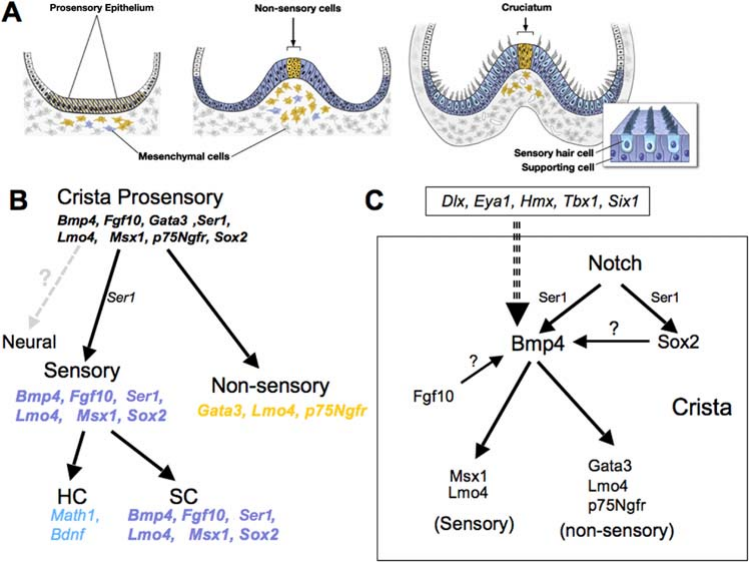
Summary of crista formation and the potential roles of Bmp4. (A) Schematic summary of crista formation from the prosensory to mature stage with sensory hair cells and supporting cells. The blue and yellow colors outside the crista represent mesenchymal *Msx1-* and *Gata3-* positive cells, respectively. Initially, a number of genes are co-expressed in the prosensory region (B), but these genes segregate into separate domains as sensory (blue) and non-sensory (yellow) fates are specified. Thus far, there is no experimental evidence that the crista prosensory region gives rise to neurons as in the prosensory regions for the maculae [48]. (C) Within the crista epithelium (box), Bmp4 expression is regulated by Notch signaling, possibly mediated by Ser1. Whether Sox2 and Fgf10 regulate Bmp4 expression is not clear. Nevertheless, Bmp4 mediates crista formation by regulating Msx1 and Lmo4 in the sensory and Gata3, Lmo4 and p75Ngfr in the non-sensory (cruciatum) regions. Furthermore, deletion of genes such as *Dlx* (*Dlx5* and *Dlx6)*, *Eya1*, *Hmx (Hmx2* and *Hmx3)*, *Tbx*, and *Six1* affects *Bmp4* expression and crista formation. Among these genes, *Tbx1* and possibly *Dlx* are co-expressed with *Bmp4* in the crista.

The developmental program for the generation of sensory patches within the vertebrate inner ear is thought to be similar to that required for sensory bristle formation in *Drosophila*, in which Notch signaling generates cell type diversity [43]–[45]. The prevailing concept is that neural fate is specified within the prosensory epithelia of the developing inner ear via Delta-Notch signaling, whereas sensory fate is maintained within the prosensory domain by positive feedback of Ser1-Notch signaling [46],[47]. Eventually, lateral inhibition mediated by Notch signaling dictates that cells within the sensory patches differentiate into either hair cells or supporting cells. There is no direct evidence for neural fate specification in the crista-prosensory regions (Figure 9). However, based on our gene expression data, we propose that at least within the prosensory domain of anterior and posterior cristae, there is an additional step of commitment into a non-sensory fate. This occurs before or at approximately the same time that hair cell and supporting cell fates are being specified. More importantly, we propose that Bmp4 is required for both specification steps by regulating Msx1 and Lmo4 activities for the sensory fate, and Gata3, p75Ngfr, and Lmo4 for the non-sensory fate (Figure 9).

Similar to the cristae but in contrast to the sensory maculae, it is not clear whether there is a specification of neural fate in the prosensory region of the organ of Corti [48]. Notably, in the organ of Corti, there are two rows of p75Ngfr-positive pillar cells that are specialized non-sensory cells located between the one row of inner and the first row of outer hair cells [49]. Interestingly, p75Ngfr is also broadly expressed in the prospective organ of Corti initially, and its expression becomes restricted to the pillar cells at later stages [50]. Thus, it is likely that similar cell fate decisions proposed here for the cristae also apply to the organ of Corti.

### *Bmp4* in Crista Formation

The requirement of *Bmp4* for crista formation is clearly indicated by results obtained from *Bmp4* conditional null mutants. Our down-regulation of Bmp signaling in presumptive cristae of chicken embryos reveals several interesting insights concerning the possible roles of Bmp4 in crista formation. First, genes that are known to be required for prosensory formation in the inner ear such as *Sox2*, *Jag1*, and *Fgf10* are not affected by either *pSmad6* (cell-autonomous) or *pNoggin* (non-cell autonomous) treatments [51]–[55]. Determining whether these prosensory genes function in parallel or directly upstream of *Bmp4* will require further investigation (Figure 9C). Second, genes in both sensory and non-sensory pathways are affected by down-regulating Bmp4 signaling. Consistent with the gene expression changes, the long-term effects are disruption of the crista structure in addition to the loss of sensory hair cells. Taken together, our results suggest that Bmp4 has a global role in organizing the structure of the crista into sensory and non-sensory domains rather than just promoting or inhibiting hair cell fate [24],[25]. This organizing role could involve interacting with the Notch signaling pathway in cell type specification.

Within the sensory pathway, both *Msx1* and *Lmo4* are affected by the reduction of Bmp signaling. *Msx1* has been shown to be downstream of Bmp4 in several other tissues [56],[57]. A similar relationship has also been suggested in the inner ear [21],[25]. No crista phenotype in *Msx1* null mutants has been reported so far, but there could be functional redundancy between *Msx1* and *Msx2* [58]. *Lmo4* is one of the Lim domain-only containing genes expressed in the inner ear [59] and is thought to be required for crista and canal formation in mice as well (Lin Gan, unpublished results). Therefore, both *Msx1* and *Lmo4* could be important mediators of Bmp4 signaling. Notably, *pNoggin* treatments appear to down-regulate these genes more effectively than *pSmad6*, presumably due to the more extensive and/or non-cell autonomous effects of Noggin.

In addition to regulating *Msx1* and *Lmo4* in the sensory region, Bmp4 could also mediate the formation of the non-sensory cruciatum by regulating *p75Ngfr, Gata3*, and *Lmo4* activities. The expression of *p75Ngfr* in the developing cristae has been known for a while [19], yet it is not clear if there is a crista phenotype in *p75Ngfr* null mutants [60].

*Gata3* is an important gene in inner ear development as evident by the rudimentary inner ear structure of *Gata3-/-* mouse embryos [31],[61]. In humans, mutations in *GATA3* are associated with HDR (hypoparathyroidism, sensorineural deafness, and renal anomaly) syndrome [62]. Given the importance of a GATA factor (pannier) in activating the achaete-scute proneural complex in *Drosophila* [63],[64], *Gata3* may have a more global effect on cell fate specification in vertebrate crista beyond formation of the cruciatum (see below). Furthermore, the conserved *Gata3* expression in the mesenchyme surrounding the presumptive cristae between chicken and mouse [65], may also contribute to the proper formation of the crista. We speculate that the observed down-regulation of *Gata3* expression in the mesenchyme by *pNoggin,* but not by *pSmad6,* may contribute to the more severe phenotype caused by *pNoggin*.

In other systems, Bmp and Gata pathways are thought to interact. For example, during *Drosophila* embryogenesis, *pannier* (homolog of *Gata1*) is induced by *dpp* (decapentaplegic, homolog of *Bmp4*) in the dorsal embryo [63]. Later in development, regulation of *dpp* becomes dependent on *pannier* [66]. Within the inner ear, *Gata3* expression appears to begin before that of *Bmp4*. However, the relatively normal *Bmp4* expression in *Gata3* null inner ears does not support *Gata3* functioning upstream of *Bmp4* [31]. Nevertheless, our results here show that the maintenance of *Gata3* in the cristae is dependent on *Bmp4*.

Furthermore, while we have classified genes as sensory and non-sensory in the above discussion according to their expression domains in a mature crista, their earlier developmental functions may not be limited to the cell types that they are expressed in at maturity. This notion is based on the ubiquitous expression of these genes in the presumptive anterior and posterior cristae. The lateral crista does not contain a cruciatum in either chicken or mouse [4]. Yet, *Gata3*, considered to be a non-sensory gene, is expressed in the prospective lateral crista of the mouse (Figure 3A). It is not known if *Gata3* is also expressed in the prospective lateral crista of the chicken. Nevertheless, based on the limited number of crista-associated genes analyzed here, the classic non-sensory genes appear to be more readily affected than the so-called sensory genes when Bmp signal transduction is down-regulated. Therefore, an attractive hypothesis that remains to be tested is that the non-sensory genes such as *Gata3* and *p75Ngfr* are key players in mediating the early organizing roles of Bmp4.

In addition, based on the inner ear phenotypes in *Bmp4* conditional knockout embryos, *Bmp4* is also required for the formation of the utricle and saccule. Since little expression of *Bmp4* is detected in their presumptive tissues using in situ hybridization [9], further study is needed to determine whether cristae are the source of Bmp4 that is required for their development.

### Bmp4 in Canal Formation

We have proposed that the sensory cristae may induce the formation of their associated semicircular canals [1]. *Bmp4* is strongly expressed in the presumptive cristae but not the canal pouch [9]. Therefore, the canal phenotypes of *Bmp4* conditional null mutants also lend support to the hypothesis that crista regulates canal formation. Recent fate mapping data in chicken indicate that there is a canal genesis zone located adjacent to each crista that gives rise to majority of the cells in the canal [36]. The expression domain of *Bmp2* in the canal pouch corresponds to this canal genesis zone, and experimental evidence suggests that Fgfs secreted from the presumptive cristae induce the formation of the canals by regulating the expression of *Bmp2* [36]. The absence of all three canals in *Fgf10* null mice is consistent with this hypothesis [54]. It is not clear, though, whether the effect of Bmp4 on canal formation is direct, or is indirectly mediated through Fgfs in the crista. The use of SU5402 (inhibitor of Fgf receptors) to block canal formation in chicken embryos also affects *Bmp4* expression in the crista (Chang and Wu, unpublished results). Therefore, both Fgfs and Bmp4 could be involved in mediating canal formation.

In addition to the postulated role of *Bmp2* in canal formation, *Dlx5* is also a key player in canal formation, and its activity is directly or indirectly regulated by Bmp4 as well. Even though the canal phenotypes in *Dlx5-/-* mutants are milder than those in *Bmp4* conditional mutants, the phenotypes in *Dlx5-/-; Dlx6-/-* double mutants appear to be more severe [16]. It is possible that there is a positive feedback loop between Dlx and Bmp4 in canal formation, such that Dlx proteins induce *Bmp4*, and in turn, their activities are maintained by Bmp4. It is also interesting that the expression of *Dlx5* in the canal pouch is more susceptible than *Hmx3* to the lack of *Bmp4*. Even though both *Dlx* and *Hmx* pathways are required for canal formation, regulation of these two pathways appears distinct. This notion is also supported by studies of *Gbx2*-/- and *Wnt1-/-; Wnt3a-/-* mutant embryos, in which *Dlx5* expression is down-regulated but *Hmx3* expression is relatively normal [12],[67].

### Regulators of *Bmp4* Expression

Given the importance of Bmp4 in forming the vestibular apparatus, it is not surprising that many genes such as *Dlx*, *Hmx*, *Tbx1*, *Eya1* and *Six1* regulate its activity, directly or indirectly (Figure 9C). Within the presumptive crista, *Bmp4* expression does not seem to be dependent on Bmp signaling (Figures 5 and 6). In contrast, *Bmp4* expression is thought to be maintained by Notch signaling [68]. When Notch signaling is blocked by DAPT, a gamma-secretase inhibitor, *Bmp4* expression in the crista is drastically reduced [68], whereas ectopic expression of activated Notch causes ectopic sensory patches, some of which are *Bmp4* positive [46]. This Notch signaling is thought to be mediated by Ser1. Consistently, mice with conditional knockout of *Jag1* fail to form cristae [51],[52].

Taken together, our results unequivocally demonstrate the importance of Bmp4 in patterning and cell fate specification of cristae and canals. Depending on the genetic background, some *Bmp4* +/− mice also display mild vestibular and auditory defects [35]. Given the multiple effects of Bmp4 in the inner ear, it is not surprising that paradoxical results were obtained from various in vitro experimental conditions [24],[25]. Blocking Bmp signal transduction in the presumptive crista of chicken reveals several potential pathways by which Bmp4 could mediate its functions. The relationships among the proteins regulated by Bmps including Gata3, Lmo4, Msx1, and p75Ngfr, will require further investigations. In erythroid cells, Gata1 and Lmo2 are known to directly interact and form a complex with other transcription factors [69]. Therefore, it is conceivable that activation of Bmp downstream genes in the crista also require Gata3 and Lmo4 to form a transcriptional complex. Future studies will focus on deciphering the relationships among these proteins in the formation of an important sensory organ, the crista.

## Supporting Information

Figure S1 Generation of a floxed Bmp4 allele in which loxP sites are inserted in introns 2 and 4. A FRT flanked Pgk-neo cassette was placed downstream of the 3′ loxP site and removed in vivo by crossing mice carrying the floxed Bmp4lox-Neo allele with ACTB-FLPe transgenic mice [70]. The targeting vector was electroporated into TL1 ES cells, and clones carrying the correctly targeted Bmp4loxP allele were identified by Southern blot analysis of genomic DNA digested with the XbaI restriction enzyme and injected into blastocysts. Homozygous Bmp4loxP mice on a 129/Black Swiss background are fully viable and show no obvious abnormalities. Compound mutant mice with Bmp4loxP and Bmp4Tm1 alleles show reduced viability with only 72% of the expected number of animals reaching weaning age. The surviving Bmp4loxP/Tm1 animals appear normal. The cause of the reduced viability is unknown.
(7.65 MB TIF)

Table S1 Summary of phenotypes.
(0.04 MB DOC)
