# Supplementary figures and images for: Bmp4 Is Essential for the Formation of the Vestibular Apparatus that Detects Angular Head Movements
Source: PLoS Genet. 2008 Apr 11;4(4):e1000050. doi: 10.1371/journal.pgen.1000050 (PMC2274953; doi:10.1371/journal.pgen.1000050)

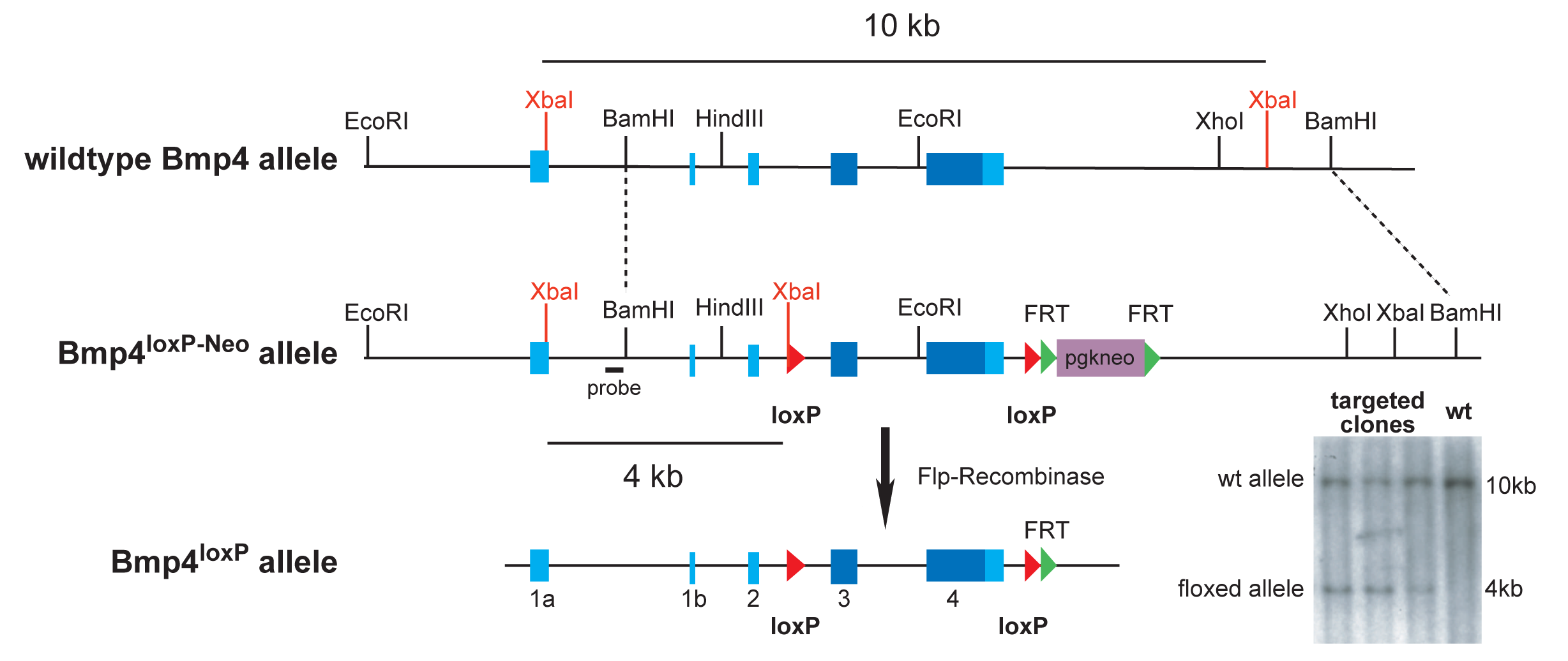

Supplement: Figure S1 — Generation of a floxed Bmp4 allele in which loxP sites are inserted in introns 2 and 4. A FRT flanked Pgk-neo cassette was placed downstream of the 3′ loxP site and removed in vivo by crossing mice carrying the floxed Bmp4lox-Neo allele with ACTB-FLPe transgenic mice [70]. The targeting vector was electroporated into TL1 ES cells, and clones carrying the correctly targeted Bmp4loxP allele were identified by Southern blot analysis of genomic DNA digested with the XbaI restriction enzyme and injected into blastocysts. Homozygous Bmp4loxP mice on a 129/Black Swiss background are fully viable and show no obvious abnormalities. Compound mutant mice with Bmp4loxP and Bmp4Tm1 alleles show reduced viability with only 72% of the expected number of animals reaching weaning age. The surviving Bmp4loxP/Tm1 animals appear normal. The cause of the reduced viability is unknown. (7.65 MB TIF) [file pgen.1000050.s001.tif]
