# Supplementary material for: Bmp4 Is Essential for the Formation of the Vestibular Apparatus that Detects Angular Head Movements
Source: PLoS Genet. 2008 Apr 11;4(4):e1000050. doi: 10.1371/journal.pgen.1000050 (PMC2274953; doi:10.1371/journal.pgen.1000050)
Supplement: Table S1 — Summary of phenotypes. (0.04 MB DOC) [file pgen.1000050.s002.doc]

Supplemental Table 1 Summary of phenotypes of *Foxg1^cre/+^*; *Bmp4^loxP/Tm1^* embryos collected between 9.5 dpc to postnatal ages

| Age | # # of  litters | # of  embryos | Average  litter size | *Foxg1^cre/+^*; *Bmp4^loxP/Tm1^* embryos ** | | | |
| --- | --- | --- | --- | --- | --- | --- | --- |
|  |  |  |  | Total #  mutants | # severely malformed | # Eye phenotype | # normal morphology |
| 9.5 dpc | 9 | 84 | 9 | 19 | 2 (11%) | 3 (16%) | 14 (74%) |
| 10.5 dpc | 10 | 91 | 9 | 24 | 5 (21%) | 12 (50%) | 7 (29%) |
| 11.5 dpc | 34 | 269 | 8 | 77 | 24 (31%) | 42 (54%) | 11 (14%) |
| 1112-13.5 dpc | 11 | 83 | 8 | 25 | 7 (28%) | 9 (36%) | 9 (36%) |
| P0 – P25 | 9 | 57 | 6* | 2 | 0 | 0 | 2 (100%) |

* Average litter size is significantly smaller than at 11.5 dpc, P < 0.05

** Numbers in brackets represent the percentage of mutant embryos with a specific phenotype
